# Supplementary figures and images for: Ruminal Fluid Transplantation Accelerates Rumen Microbial Remodeling and Improves Feed Efficiency in Yaks
Source: Microorganisms. 2023 Jul 31;11(8):1964. doi: 10.3390/microorganisms11081964 (PMC10458777; doi:10.3390/microorganisms11081964)

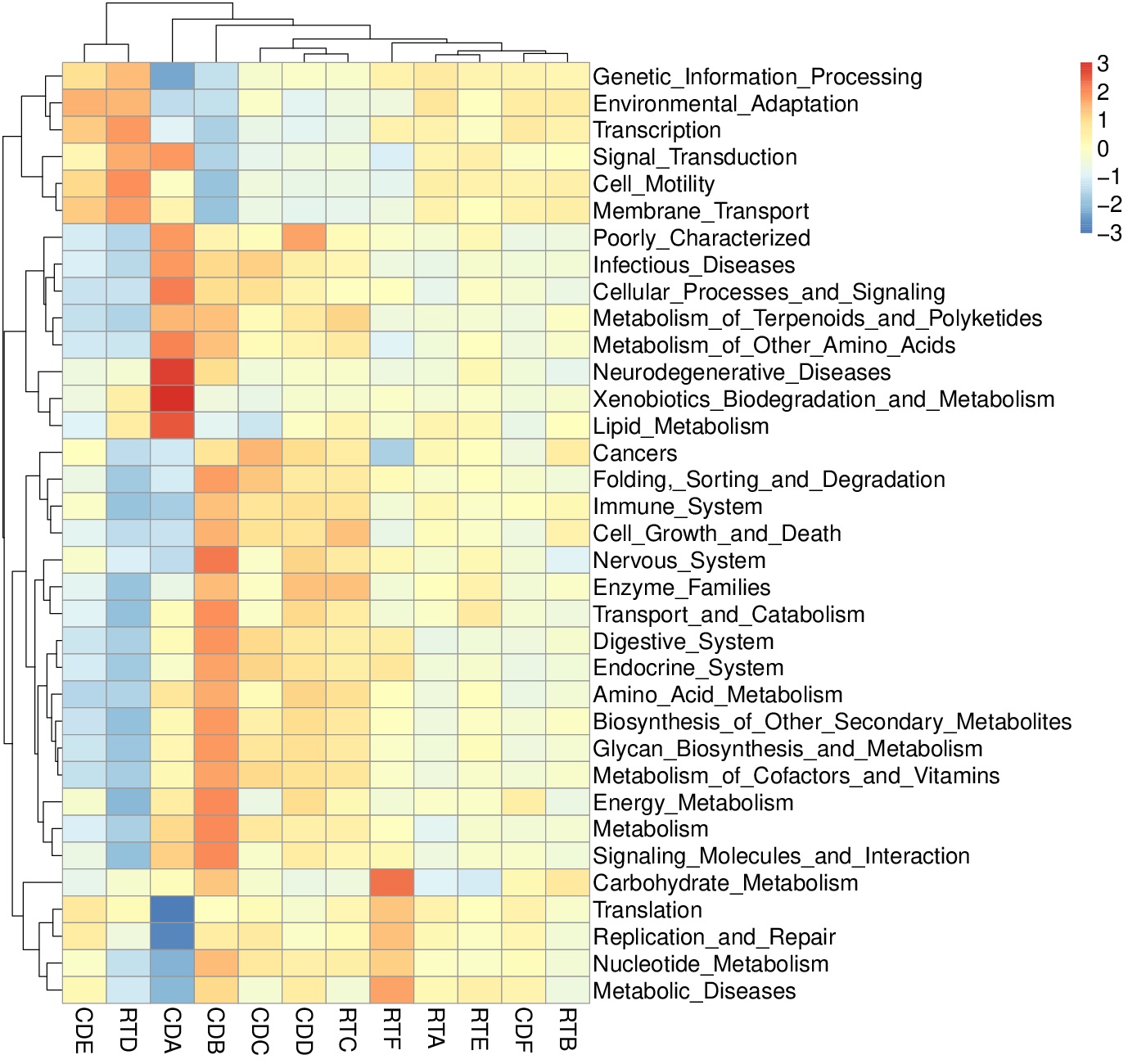

Supplement: Supplementary file 1 [file microorganisms-11-01964-s001.zip › microorganisms-2458172-supplementary.jpg]
